# Satellite Fingerprinting Methods for GNSS Spoofing Detection

**DOI:** 10.3390/s24237698

**Published:** 2024-12-01

**Authors:** Francisco Gallardo, Antonio Pérez-Yuste, Andriy Konovaltsev

**Affiliations:** 1 ETSI Sistemas de Telecomunicación, Universidad Politécnica de Madrid, 28031 Madrid, Spain; antonio.perez@upm.es; 2 DLR GfR mbH, 82234 Weßling, Germany; 3 Institute of Communications and Navigation, German Aerospace Center (DLR), 51147 Köln, Germany; andriy.konovaltsev@dlr.de

**Keywords:** satellites, global navigation satellite system, Galileo, SCER, machine learning, estimation, satellite fingerprinting

## Abstract

Spoofing attacks pose a significant security risk for organizations and systems relying on global navigation satellite systems (GNSS) for their operations. While the existing spoofing detection methods have shown some effectiveness, these can be vulnerable to certain attacks, such as secure code estimation and replay (SCER) attacks, among others.This paper analyzes the potential of satellite fingerprinting methods for GNSS spoofing detection and benchmarks their performance using real (in realistic scenarios by using GPS and Galileo signals generated and recorded in the advanced GNSS simulation facility of DLR) GNSS signals and scenarios. Our results show that our proposed fingerprinting methods can improve the detection accuracy of the existing methods and can be coupled with other techniques to enhance the overall performance of the detection systems, all based on relatively simple metrics. In this paper, we compare the performance of several fingerprinting methods, including those from the existing literature (based on signal Gaussian properties of the signal complex envelope, energy and in-phase symbol dispersion) and one proposed in this paper, based on the satellite instrumental delay. The innovation of this work is a new jamming and spoofing complementary detection technique, based on fingerprinting and machine learning, including a new fingerprinting metric (based on the satellite instrumental delay).

## 1. Introduction

Global navigation satellite systems (GNSS) are crucial in many applications, including navigation, surveying, and timing. Nonetheless, they are vulnerable to jamming and spoofing attacks. Spoofing attacks are those where an attacker generates false signals that can deceive the receiver and lead to providing incorrect position or timing information. Secure code estimation and replay (SCER) attacks are one of the most challenging types of spoofing attacks. These can be difficult to detect due to the usage of the real navigation message [1].

Researchers have recently proposed various methods for detecting and mitigating spoofing attacks [2]. However, the existing methods can be vulnerable to certain types of attacks, such as the SCER attacks, and may not be effective in all scenarios. Therefore, complementary protection techniques are necessary to enhance the security and reliability of GNSS receivers.

In this paper, we propose a new approach to protect GNSS receivers against spoofing attacks, based on satellite fingerprinting. Satellite fingerprinting methods involve extracting unique features from GNSS signals to identify the satellite transmitting source and distinguish between genuine and spoofed signals. We benchmark the performance of a system based on several fingerprinting methods fed to machine learning algorithms, using realistic GNSS signals and scenarios to compare them to the existing methods.

Our proposed approach builds on the insights gained from our previous work on SCER attacks on Galileo systems [3] and the development of advanced antenna arrays for jamming and spoofing detection [4]. We analyze the role of satellite fingerprinting in enhancing the performance of existing detection methods and discussing the trade-offs and applicability of different fingerprinting solutions. Our results show that satellite fingerprinting methods are promising for improving the detection accuracy of the existing protection subsystems and can be coupled with other techniques to enhance the overall performance of the detection system.

### 1.1. Secure Code Estimation and Replay (SCER) Spoofing Attacks

The challenge posed by SCER attacks is that these involve the spoofer embedding the authentic navigation message within a counterfeit signal while adding differential delays and/or Doppler shifts to each satellite signal. This manipulation causes the target to compute incorrect position, velocity, and/or time information.

These attacks rely on real-time symbol estimation by the attacker, who applies Bayesian estimators to the output of a matched filter, as described in [1].

Since SCER attacks use authentic navigation messages, solutions based solely on digital signatures within the navigation message (e.g., OS-NMA) may be ineffective. This highlights the need for complementary detection methods.

The existing techniques have been developed specifically to counter SCER attacks; however, they often require high bandwidth and are computationally intensive, such as the approach in [3], which was primarily designed to protect critical infrastructure. Therefore, more adaptable and resource-efficient solutions are necessary.

### 1.2. Overview of Existing State-of-the-Art Methods

Authors in [5] analyzed the usage of SVMs (Support Vector Machines) to detect the presence of spoofers in the received signals, based on IQ imbalances, analog to digital converters (ADC) non-linearities, phase-noise and power amplifier non-linearities. Their work expanded the one proposed in [6], where the system and metrics were tested with signals with $C/N_{0}$ of 100 dBHz (based on simulations) only. In [6], the authors obtained 100% accuracy, while in [5], with a more realistic setup (signals recorded in three different EU sites, with non-homogeneous HW across sites), the obtained accuracy was 99.99% for pre-correlation metrics and 87.72% for post-correlation metrics.

In [7], authors discuss fingerprinting features and the usage of multivariate normal distribution (MVN) to predict the presence of spoofers. The usage of K-folds for the correct cross-validation is highlighted, which helps to better understand the produced solution (“Spotr” system) generalization. These authors also used the UT-Austin TexBat for testing their solution. It is important to highlight that the authors achieved both a false positive rate (FPR) and a false negative rate (FNR) of 0% simultaneously. This indicates that the system was able to classify the entire dataset correctly. In the best-case scenario, the testing dataset comprised 35,000 data points. Therefore, the accuracy of the Spotr system shall be better than $1-error_{SPOTR}>1-\frac{1}{35000}=0.99997$. However, since the authors did not provide the confusion matrix for the results or specify whether the dataset was balanced, it is not possible to infer the probability of false alarms or missed detections.

In [8], the authors propose two methods, one implying the usage of two-stage artificial neural networks (TS-ANN) and K-nearest neighbors (K-NN) based on signal quality monitoring (SQM) metrics and another one, using variational auto-encoders (VAE). The authors assessed the proposed methods using the TexBat dataset, providing remarkable results in the range of 0.49% for PFA and PMD of 0.39%. The authors also point out that the VAE methods are only trained with spoofing-free data, theoretically being able to detect patterns that deviate from authentic data. That last point could be of great further applicability not only for finding spoofing attacks in the received signals but also anomalies in satellites and receivers, which may be an interesting and natural expansion of the authors’ methodology.

Researchers in [9] proposed a method based on deep learning (RESNET50) and short-time Fourier transform (STFT), providing an accuracy of 53.9% and a convolutional neural network (CNN) directly fed with IQ signal samples, providing an accuracy of 65%. Authors in [10] fed a VAE with the GNSS receiver search space, obtaining an accuracy of 95.68%.

An accuracy of 99.7% was achieved in [11] by applying a convolutional auto-encoder (CAE) and a noise-like signal reconstruction.

Table 1 provides the list of state-of-the-art methods for spoofing detection that use fingerprinting and machine learning techniques. The column “Reported accuracy” represents the ratio of correct classifications made by the algorithms to the total number of samples.

Table 8, in Section 3.9, provides metrics that allow the comparison of both the state-of-the-art solutions and the proposed technique.

Although these results are remarkable, some applications and markets (for instance the defense or aeronautical markets) require very high accuracy, low probability of false alarm (PFA) and low probability of missed detection (PMD). To enhance the accuracy of the existing methods, we propose the integration of a novel fingerprinting metric leveraging satellite differential delays. This metric is fused with established fingerprinting metrics. Subsequently, we conduct comprehensive testing employing various artificial intelligence (AI) techniques. These tests were conducted within realistic scenarios utilizing GPS and Galileo signals. These signals were generated and recorded using the advanced GNSS simulation facility of Deutsches Zentrum für Luft und Raumfahrt (DLR).

The remainder of the paper is organized as follows. In Section 2, we introduce the used fingerprinting algorithms. Section 3 discusses the test setup, provides the results and compares them to the ones obtained by the state-of-the-art work (which was introduced in this section). In Section 4, we conclude the paper by discussing the implications of our findings and highlighting future research directions.

## 2. Used Fingerprinting Metrics

The used metrics are based on the analysis of several metrics across the GNSS receiver blocks. These metrics comprise a fusion of both pre-correlation and post-correlation metrics, with certain metrics tailored specifically for the detection of radio frequency interferences (RFIs), which sometimes precede a spoofing attack [3]. These metrics are as follows:

1. Satellite instrumental delay-based metric.
2. In-phase symbol dispersion at the tracking stage.
3. Gaussian properties of the Galileo signal complex envelope: kurtosis and skew.
4. Teager–Kaiser energy operator (TKEO).

The metric depending on the satellite instrumental delay is a novelty compared to the state of the art related to spoofing detection. The new proposed metric is the one presented in Section 2.1.1.

### 2.1. New Proposed Metric

In this section, the new proposed metric based on the satellite instrumental delay is explained.

As detailed further in Section 2.1.1, this metric is proposed for spoofing detection because a spoofer may leave detectable traces in the fake generated signal, especially if the spoofer’s electronics exhibit significantly different behavior compared to authentic satellites. Such traces can occur in the following cases:

- The spoofer fails to control the delay between channels and/or the coherency between code and phase.
- The spoofer performs a single-frequency attack.

#### 2.1.1. Satellite Instrumental Delay

The GNSS signals that are received by user terminals suffer dispersive effects that cause some differential delays between signals at different frequencies (e.g., E1 compared to E5). Such differential delays are caused by the satellite’s and the receiver’s electronics, as well as by atmospheric dispersive effects.

By estimating the differential satellite delay (or, to be more precise, deriving a metric that depends directly on the satellite differential delays), another fingerprinting feature can be included in the machine learning algorithms used for spoofing detection.

In order to analyze the typical values of instrumental differential delay that a GNSS satellite may have, a useful tool is the broadcast group delay (BGD).

The definition of BGD (Galileo) is the one in Equation (1), as indicated in [12].

(1) $$BGD\left(f_{1},f_{2}\right)=\frac{TR_{1}-TR_{2}}{1-{\left(\frac{f_{1}}{f_{2}}\right)}^{2}}$$

where $TR_{1}$ and $TR_{2}$ are the group delays in frequency 1 and 2, and $f_{1}$ and $f_{2}$ are the frequencies 1 and 2, respectively.

It shall be noted that the BGD corrections transmitted in the navigation message are referred (scaled) to the Iono-free combination, and therefore, the comparison is not direct to the proposed metric, which is based on delays only. Nonetheless, it helps to understand the range of differential delay values that one could expect from a real satellite. Galileo transmits in its navigation messages $BGD\left(E5a,E1\right)$ and $BGD\left(E5b,E1B\right)$. Indeed, these corrections shall be applied to clock corrections transmitted by Galileo in case the end-user is only working with a single frequency. Therefore, an analysis of all the Galileo constellation, for E5a/E1b and E5b/E1b, for all 2022, was first performed.

In order to extract the BGD(E1, E5a) information from the Galileo navigation message, the data are obtained from page type 1 of the subframes of the F/NAV navigation messages. The parameters BGD(E1, E5a) and BGD(E1, E5b) can be found in the words type 5 in the I/NAV navigation messages; further information can be found in [12].

**Figure 1:**
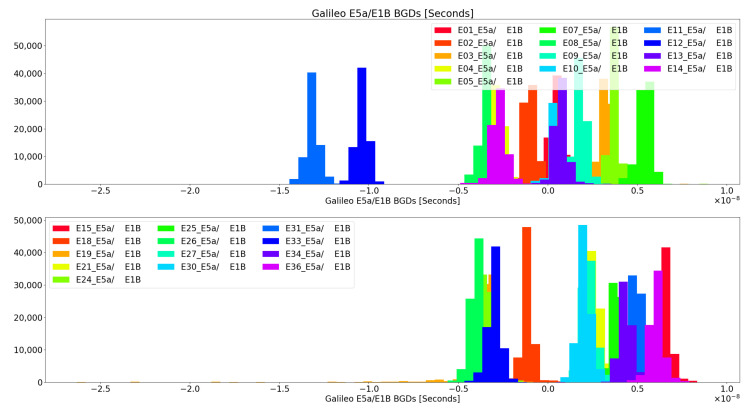
Broadcasted E5A/E1B BGDs for all Galileo Constellation across 2022.

**Figure 2:**
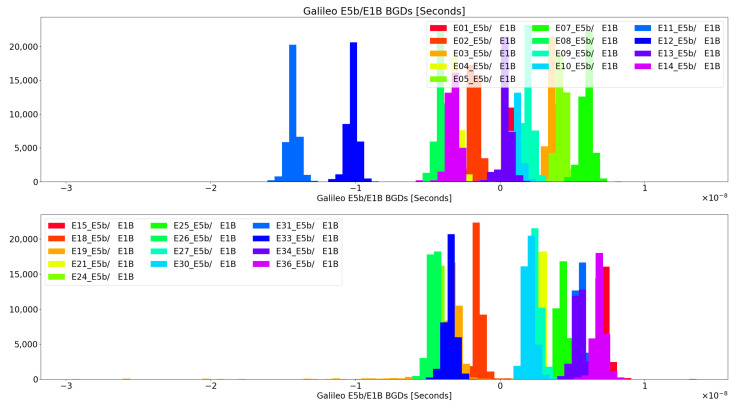
Broadcasted E5B/E1B BGDs for all Galileo Constellation across 2022.

All the past navigation messages transmitted by the different GNSS constellations are available in the International GNSS Service (IGS) monitoring network archives. We used all the yearly data for 2022 and processed all the BGD values for E5a/E1b and E5b/E1b. The histograms of such values can be found in Figure 1 and Figure 2. The great majority of the BGD values for E5a/E1B and E5b/E1B are within −5ns and +7ns, except for the values of E12 and E11, which happen to be the oldest Galileo satellites still in service. This also implies the interest of monitoring the historical trends in BGD values, from a user perspective, to detect and correct possible mission segment anomalies and/or to detect intents of spoofed signals transmission, as this may be an indication that the hardware (HW) transmitting the signals does not present the same characteristics of the real satellites at any given time (i.e., same HW units and same aging status). Other authors already pointed out differences in these BGD values for the Galileo in orbit validation (IOV) satellites in the past [13]. Moreover, differences in the group delay among the IOV satellites are reported in the Galileo metadata, available from the GNSS Service Centre website [14], being both the satellite group delay measured on ground (prior to the launch) and the differential code bias (DCB), considerably bigger for E11 and E12 (GSAT0101 and GSAT0102) than for the other two IOVs E19 and E20 (GSAT0103 and GSAT0104). Note that E20 is no longer providing service, so its data cannot be found in the 2022 provided report. As these differences were already present in the satellites metadata prior to their launch, the source of these behaviors cannot be related to aging and it could simply be related to the payload setup, hence, indicating the relevance of fingerprinting monitoring for spoofing detection, as in this case, the attackers would need to keep a table with the current BGD values per satellite and signal, and emulate those values.

Considering the probability distribution functions (PDF) outlined in Figure 1 and Figure 2, these could also be used for determining how likely (a priori knowledge) it is that a particular BGD metric measured value could be produced by a satellite or not.

In order to derive a metric that depends on the group and phase delay, we will follow the algorithm outlined in [15]; particularly, we will

1. Compute the pseudo ranges in two frequencies (e.g., E1 and E5) and calculate the geometry-free combination both from code (Equation (2)) and phase (Equation (3)).
  (2) $$PI_{i}^{j}=P2_{i}^{j}-P1_{i}^{j}$$
  (3) $$LI_{i}^{j}=L1_{i}^{j}-L2_{i}^{j}$$
  Here, $P1_{i}^{j}$ and $P2_{i}^{j}$ represent the pseudo-range estimates (code) between receiver *i* and satellite *j* at frequencies 1 and 2, respectively. Similarly, $L1_{i}^{j}$ and $L2_{i}^{j}$ denote the pseudo-range estimates (phase) for the same receiver–satellite pair at frequencies 1 and 2, respectively.
2. Then, we will estimate the undifferentiated carrier-phase ambiguity-free combination (BI) from the averaged difference value of the code and the phase geometry-free combination for an entire tracked satellite arc, as indicated in [15].

(4) $${\bar{BI}}_{i}^{j}\approx <PI_{i}^{j}-LI_{i}^{j}{>}_{arc}$$

where $BI_{i}^{j}$ is the undifferentiated carrier-phase ambiguity-free combination between satellite “*j*” and receiver “*i*”. PI is the geometry-free pseudo-range code combination, and LI is the geometry-free pseudo-range phase combination. The operator $<{>}_{arc}$ means averaging over the tracked arc for a particular GNSS satellite that the victim receiver is tracking.

Note that as the methodology for the BI estimate relies on an entire tracked arc, it makes the application of this metric cumbersome for real time, making the best approach a continuous estimate of the BI value with the available data.

By analyzing this expression we can obtain Equation (5).

(5) $${\bar{BI}}_{i}^{j}\approx b_{E5}^{j}-b_{E1}^{j}+k_{E5}^{j}-k_{E1}^{j}$$

where $b_{E5}^{j}$ and $b_{E1}^{j}$ are the satellite group delays per frequency (Galileo E1 and E5), and $k_{E5}^{j}$ and $k_{E1}^{j}$ are the phase satellite delays per frequency. In order to obtain this result, it is necessary to assume the process noise is such that its expectation is 0, the wind-up effect shall be corrected, the carrier-phase integer ambiguity shall be corrected (e.g., by leveling carrier and phase, as in [16]), and it is necessary to provide the receiver, computing the GNSS observables, with a calibration signal (both in E1 and E5 bands) to measure the additional group delay and carrier phase rotations introduced by the receiver hardware. These assumptions can be considered realistic, as these types of computations are performed in professional high-grade GNSS receivers. In future work, during the prototype development, the engine to compute such parameters in a realistic scenario will be implemented.

Rearranging Equation (5) leads to Equation (6).

(6) $${\bar{BI}}_{i}^{j}\approx \Upsilon_{Group_{Sat}}+\Lambda_{Phase_{Sat}}$$

where $\Upsilon_{Group_{Sat}}=b_{E5}^{j}-b_{E1}^{j}$ and $\Lambda_{Phase_{Sat}}=k_{E5}^{j}-k_{E1}^{j}$.

Due to the way the computation of the undifferentiated carrier-phase ambiguity-free combination (BI) is being performed, the estimate and the feature are not readily computed in real-time. It will be computed with some delay and only for those satellites where the tracking was long and continuous enough to obtain an arc with sufficient length. For real-time computation, the algorithm shall be modified, potentially using more advanced solutions for ambiguity resolution, like those used in precise point positioning (PPP). If the spoofing attack takes several minutes, such convergence may be possible [17], detecting attacks extending for more than four minutes.

Once the computation of the satellite group and phase delays is outlined, two scenarios will be analyzed to understand the theoretical impact that a spoofing attack would have in such a metric:

1. The spoofer is making a dual-frequency spoofing attack (e.g., E1 and E5)
2. The spoofer is making a single-frequency attack (e.g., E1 alone).

##### Dual-Frequency Spoofing Attack

As the SCER spoofer attack is based on adding additional delays to the original signals (as it is assumed that the navigation message shall remain the real one in the case of the presence of the navigation message authentication (NMA) like the Galileo OS-NMA [18]), the spoofer will eventually impact the pseudo-range measured by the victim. In this example, the spoofer would be making a dual-frequency spoofing attack (i.e., both fake E1 and E5 signals will be generated by the spoofer).

By considering the spoofer influence in the pseudoranges in Equation (6) and dropping the satellite and receiver indexes for simplicity, we obtain

(7) $${\bar{BI}}_{i}^{j}\approx \Upsilon_{Group_{Sat}}+\Lambda_{Phase_{Sat}}+\epsilon_{Code}^{Spoofer}+\epsilon_{Phase}^{Spoofer}$$

where $\epsilon_{Code}^{Spoofer}=\Theta_{Code_{5}}-\Theta_{Code_{1}}$ and $\epsilon_{Phase}^{Spoofer}=\Theta_{Phase_{5}}-\Theta_{Phase_{1}}$. $\Theta_{Code_{1}}$ is the influence of the spoofer in the victim’s computed pseudorange in the frequency E1 for code only, $\Theta_{Code_{5}}$ is the influence of the spoofer in the victim’s computed pseudorange in the frequency E5, for code only. $\Theta_{Phase_{1}}$ is the influence of the spoofer in the victim’s computed phase in the frequency E1, and $\Theta_{Phase_{5}}$ is the influence of the spoofer in the victim’s computed phase in the frequency E5.

In order to make the effect zero, the spoofer has to ensure the influence in both frequencies (E1 and E5) is the same, taking into account that the spoofer will not want to set a different influence in code and phase (i.e., phase and code coherency are ensured by the spoofer to avoid alerting the victim), then we obtain Equation (8).

(8) $${\bar{BI}}_{i}^{j}\approx \Upsilon_{Code_{Sat}}+\Lambda_{Phase_{Sat}}+2\left(\Theta_{5}-\Theta_{1}\right)$$

where $\Theta_{5}$ is the influence of the spoofer on the pseudorange in E5 (assuming code and phase influence is the same, as discussed above), and $\Theta_{1}$ is the influence of the spoofer on the pseudorange in E1 (assuming code and phase influence is the same). It is assumed that both metrics are measured in distance units.

As seen in Equation (8), the impact on the metric will depend on the term $2\left(\Theta_{5}-\Theta_{1}\right)$. In the SCER attack, the spoofer **needs** to make these individual values different to 0. Now, if the electronics of the spoofer are good enough to keep alignment between both frequencies (i.e., the group delay, phase delay and coherency between code and phase of the spoofer electronics are under control), then no effect will be noticeable by the victims. In other words, as long as $\Theta_{1}=\Theta_{5}$, then the victims will not notice any effect.

However, if the spoofer is not able to control it, the effect in the metric will be twice the difference or will leave a trace in terms of phase/code coherency.

##### Single-Frequency Spoofing Attack

In this case, the spoofer only performs the SCER spoofing attack in a single frequency. This could be the case if the attacker does not have the means to perform a dual-frequency attack and follows a more simple and, at the same time, more feasible approach. In such cases, Equation (8) becomes Equation (9) (assuming only E1 frequency is used for the spoofing attack and assuming again the spoofer wants to keep phase/code coherency).

(9) $${\bar{BI}}_{i}^{j}\approx \Upsilon_{Group_{Sat}}+\Lambda_{Phase_{Sat}}+2\left(\Theta_{1}\right)$$

This is a very interesting result because this means that in case the spoofer does not conduct a dual-frequency attack and controls the phase and code alignment between both channels, then **the more the spoofer influences the pseudo-range computation of the victim, the more it will impact the satellite group and phase delay monitoring, which virtually kills the SCER SF attack**. Indeed, the influence will be two times the change in the pseudo-range caused by the spoofer.

For instance, this implies that a change of 500 extra meters in a pseudo-range for a particular satellite implies a 1000 m impact in the metric.

In general, if the spoofer is using cheap front-ends, it may have problems keeping the code-carrier phase coherency under control, even for a single frequency.

### 2.2. Legacy Metrics

This section discusses the legacy metrics commonly used in the literature and in this work.

#### 2.2.1. In-Phase Symbol Dispersion

This metric is based on the distortion that the spoofer may introduce in the in-phase axis, containing the transmitted symbols [19], as seen at the output of the prompt correlator in the tracking stage.

The output of the prompt correlator in the tracking stage follows Equation 1.91 in [20].

(10) $$y_{p}\left[k\right]=\sum_{n=0}^{N_{T}-1}r\left[n+kN_{T}\right]c^{*}\left[n-\hat{\tau }\right]N_{code}e^{-j\hat{\Theta }\left[n;k\right]}$$

where $e^{-j\hat{\Theta }\left[n;k\right]}$ is the locally generated carrier replica, r[n] is the received signal, $N_{code}$ is the number of samples in one code period $\left(N_{T}\right)$, and c[n] is the PRN-code local replica.

In the case of the Galileo E1B signal, if the signal is properly aligned, we will find the symbols of the navigation message in the real part of the output of the prompt correlator.

In the presence of a spoofing signal, Equation (10) becomes Equation (11).

(11) $$y_{p}\left[k\right]=\sum_{n=0}^{N_{T}-1}\left(\upsilon +\xi \right)c^{*}\left[n-\hat{\tau }\right]N_{code}e^{-j\hat{\Theta }\left[n;k\right]}$$

where $\upsilon =r[n+kN_{T}]$ and $\xi =r_{s}\left[n+kN_{T}\right]$. The spoofer received signal is indicated as $r_{s}\left[n\right]$.

Synchronous attacks [21] are those where the spoofer is able to match the phase and delay of authentic signals in the victim’s receiver. This type of implementation is a sophisticated way of performing a spoofing attack, leaving little trace in many spoofing detectors.

Nonetheless, in such a case, in the real part of the prompt correlator output, we will find a sum of the spoofing symbols and the real satellite symbol. In case of having a single signal, this being the one from the satellite or the one from the spoofer, or if the prompt correlator alignment is only correct with one of the two signals, then only a set of symbols will be found in the prompt correlator output. Such symbols will be either the one from the satellite or the one from the spoofer.

Hence, in the case of having a single signal aligned with the prompt correlator,

(12) $$Re\left\{y_{p}\left[k\right]\right\}=E1B+\eta$$

where η is the term modeling the result of the correlation of the predominantly thermal noise at the output of the receiver front-end with the local PRN-code replica. This noise term is assumed to be Gaussian.Furthermore, in case of having two,

(13) $$Re\left\{y_{p}\left[k\right]\right\}=E1B_{sat}+\eta_{sat}+E1B_{spoofer}+\eta_{spoofer}$$

Based on Equation (13), we can identify that we will find two Gaussian distributions that will be centered in the symbols transmitted by the satellite and the spoofer. The exact values, which depend on the symbols being transmitted, will also depend on the power transmitted by the satellite and the spoofer and the alignment of such signals with the prompt correlator.

As mentioned before, in case the alignment in the victim’s tracking stage only takes place with the spoofer and not the real satellite, then Equation (12) will be seen, and no distortion will be noticeable by the victim.

In the SCER synchronous attack, the attack needs to “hijack” the victim prompt correlator, then a distortion should be expected, if the attack is carried out smoothly by the spoofer, particularly if the power is different with respect to the satellite one.

During the synchronous attacks, in order to ensure the hijacking of the victim’s prompt correlator, the spoofer may slightly increase the transmitted power after the code and phase alignment is achieved. That will leave a more noticeable effect in this metric. More details on the practical implementation of this metric can be found in Section 3.5. Using ML-algorithms to detect abnormal pattern in the IQ diagrams makes them ideal for detecting this type of perturbance.

#### 2.2.2. Gaussian Properties of the Front-End Output Signal

Assuming nominal working conditions, the input, which is a signal on the intermediate frequency, to the ADC in the GNSS receiver should be dominated by Gaussian noise [3]. As such, the presence of a spoofer shall not change that, as the GNSS spreaded signals show Gaussian properties and should be below the noise level. Nonetheless, it is widely known that some attackers start a spoofing attack by injecting a jamming signal prior to the start of the attack [1], mainly to force a re-acquisition of the signals by the victim receiver, when the spoofing signals are being transmitted. This is why some techniques actually look, in a parallel manner to the spoofer detection, for interferences [3]. In this case, we will look at the Gaussian properties of the signal in the ADC input as that can also be signaling an attack.

The signal model at the ADC input follows [3]:

(14) $$Y_{GNSS-IF}=GNSS_{signal}cos\left(2\pi f_{IF}t+\theta \right)+N$$

where $GNSS_{signal}$ is either the Galileo signal in the E1 (or E5) band or the GPS signal in the L1 band (or L5), $f_{IF}$ is the intermediate frequency after downconverting the RF signal, θ is the initial phase of the IF carrier, and *N* is the noise term in the ADC input. $N\approx N\left(0,\sigma^{2}\right)$.

The detection of the jamming signals is key, some authors are even proposing methods to suppress such jamming signals to enhance the spoofing detection, [22]. Although this correction is not implemented in our methodology, this alternative could help reduce false alarms.

##### Skew

The Fisher–Pearson coefficient of skewness is defined as Equation (15) [23].

(15) $$g_{1}=\frac{m_{3}}{m_{2}^{3/2}}$$

where $m_{3}$ and $m_{2}$, are the third and second moments [23].

(16) $$m_{i}=\frac{1}{N}\sum_{n=1}^{N}{\left(x\left[n\right]-\bar{x}\right)}^{i}$$

where x[n] is the complex envelope-based band signal that was received by the victim antenna, down-converted and digitized. The skewness measures how symmetrical [24] is the probability distribution of the noise. Being a normal distribution, centered in zero, the skewness is expected to be very low, close to zero. In an ideal case, it should be zero (when the signal is in base-band).

##### Kurtosis

While the skewness measurement tells us about the symmetry of the probability distribution function (PDF), the kurtosis will provide us information on the “peakness” of the PDF. In our case, it will be linked to the noise in the input of the ADC.

In order to provide a normalized measurement, we will use Fisher’s definition of kurtosis. This implies that 3.0 is subtracted from the result to give 0.0 for a normal distribution, see Equation (18). This is known as the excess of kurtosis. Pearson’s and Fisher’s definition can be found in Equations (17) and (18), respectively, both from [23].

As we are going to use Fisher’s definition, in a nominal situation (i.e., no jamming signals in the ADC input), we should expect values close to 0 for the kurtosis metric.

(17) $$g_{2}=\frac{m_{4}}{m_{2}^{2}}$$

(18) $$g_{2}-3=\frac{m_{4}}{m_{2}^{2}}-3$$

where $m_{2}$ and $m_{4}$ are the second and fourth moments, following Equation (16).

Finally it is to be noted that even with GNSS-like signals, if the attacker used power such that the victim’s ADC is saturated, the Gaussian properties may also be lost.

#### 2.2.3. TKEO

The Teager–Kaiser energy operator (TKEO) metric is widely used in medical signal processing and was suggested for spoofing detection in [6].

This energy operator will allow us to detect when signal energy increases unexpectedly and will be used as another metric for detection. The aim of the usage of this metric is to detect unexplainable increases in energy that may be related to the presence of either strong GNSS-like signals or, most likely, jamming signals that may precede a spoofing attack. It will be applied to IQ samples in the raw signal processing blocks.

The TKEO operator is defined as in Equation (19), as seen in [25].

(19) $$\phi \left[x[n]\right]=\left(x_{r}^{2}\left[n\right]-x_{r}\left[n+1\right]x_{r}\left[n-1\right]\right)+\left(x_{i}^{2}\left[n\right]-x_{i}\left[n+1\right]x_{i}\left[n-1\right]\right)$$

where x[n] is the complex base band signal that was received by the victim antenna, down-converted and digitized. The real part of x[n] is $x_{r}\left[n\right]$, and $x_{i}\left[n\right]$ is the imaginary part.

It is to be noted that the operator is non-causal, implying that in order to operate in “real-time”, it will be required to operate with some delay (at least one sample). The TKEO operator will provide low values if the amplitude of the signal remains constant (indeed, it will provide a zero output if the signal remains constant in the current, last and next samples).

In the case of overpowered spoofing attacks, and/or in case the spoofer is using powerful jamming signals at the beginning of an attack, we will see an unusual increase in the values of the energy operator, at least during the amplitude transitioning periods.

## 3. Test Setup

### 3.1. The Fingerprinting Testbed

A Python (version 3.10.12) testbed was developed in order to test the concept proposed in this paper.

**Figure 3:**
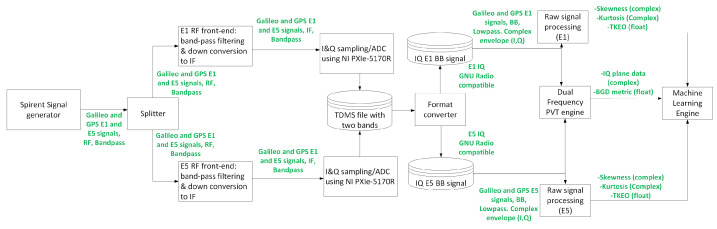
Test setup, including the MASTER facility and the SW-based (python) testbed.

The overall testbed, comprising the MASTER facility (including the Spirent signal generator, splitter and the front-end for digitization and signal file generation) and the software-based test system (including the format converter, two raw signal processing blocks, dual-frequency PVT engine and machine learning engine), is illustrated in Figure 3.

This testbed is not designed to run in real time but to test the performance of the proposed metrics for spoofing and jamming detection. It is divided into the following blocks:

- The MASTER facility (including the Spirent signal generator, the splitter and the front-end).
- Format converter.
- Raw signal processing.
- Dual frequency PVT engine.
- Machine learning block.

### 3.2. DLR GNSS Laboratory: MASTER Facility

**Figure 4:**
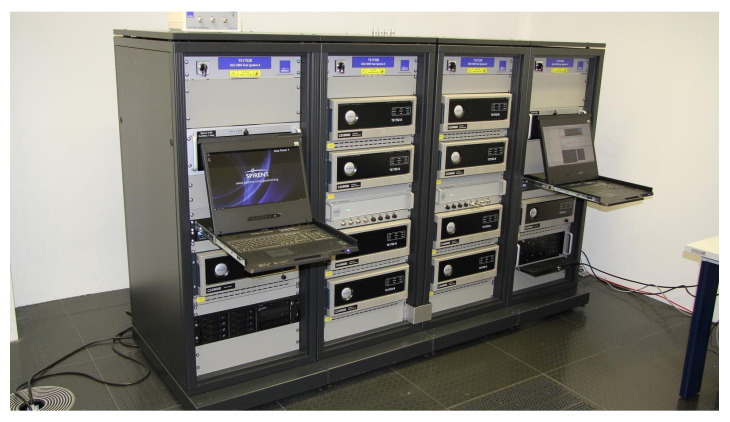
DLR Multi-output Advanced Signal Test Environment for Receivers (MASTER).

The MASTER facility, which can be seen in Figure 4, is the large-scale multi-output advanced signal test environment for receivers. It is situated at the DLR Institute of Communications and Navigation within the German Aerospace Center (DLR) in Oberpfaffenhofen (Germany). It serves as a distinctive and potent hardware simulation tool, specifically designed for evaluating and testing GNSS receivers under realistic signal conditions. This simulator is composed of eight Spirent GSS9000 units and offers signals from various global navigation systems, including GPS, Galileo, GLONASS, and BeiDou, along with regional augmentation systems such as quasi-zenith satellite system (QZSS), wide area augmentation system (WAAS), and European geostationary navigation overlay service (EGNOS).

Users have complete autonomy over the specification of satellite orbit, as well as time and date settings. Furthermore, key parameters affecting signal quality, such as orbit and timing errors, ionosphere and troposphere signal propagation delays, multipath propagation, and interference and spoofing, can be finely adjusted. Specific signal components, including data and pilot channels, navigation messages, and spread codes, can be individually selected.

The MASTER platform also allows the generation of sophisticated scenarios with all kinds of GNSS spoofing attacks, which were used for the current research.

The splitter divides the E1/E5 signals generated by MASTER into two in-house built E1 and E5 RF front-ends. These front-ends developed by DLR perform band-pass filtering and down-convert to an intermediate frequency (IF) of 75 MHz. Then, an oscilloscope board PXIe-5170R of National Instruments (National Instruments Corporation, Austin, TX, USA) is used to sample the front-end E1 and E5 outputs. The board has four channels (only two are actually used in the test set-up) where the signals are sampled with 14-bit ADCs at a 100 MHz sampling rate. The digitized signals are further filtered (in the SW running in the oscilloscope board) and decimated to 20 MHz and then converted to base-band. The resulting I&Q samples for both frequency bands are stored in a single binary file in technical data management system (TDSM) format.

### 3.3. Format Converter

As the NI board that digitizes the signal generates dual-frequency TDMS files, a basic format converter was included in the testbed. Such a format converter reads the generated dual-frequency (E1 and E5) TDMS files and converts them into single-frequency 16 bit IQ files (compatible with GNU radio and GNSS software defined radio (SDR)). This means that from each TDMS file generated by the NI board, two separate files (one for E1, another one for E5) are generated, both with the same sampling frequency of the original file (20 MHz).

### 3.4. Raw Signal Processing Block

**Figure 5:**
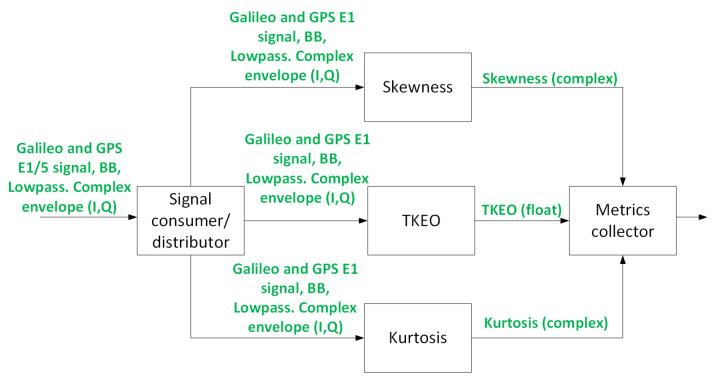
Raw signal processing block.

This block implements the metrics of TKEO and front-end skew and kurtosis. As depicted in Figure 5.

Two of these blocks are required, one for E1 and another for E5. The analysis window for all the metrics computed in the raw signal processing block is 4 ms, which means that every 4 ms, there will be a metric to collect from each one of the sub-blocks of the raw signal processing block. Merging the metrics and reporting them to the upper logic block is the task of the “Metrics collector” block.

### 3.5. PVT Engine

This block is in charge of computing the pseudoranges used for the $\bar{BI}$ metric computation, see Section 2.1.1 and the IQ planes analysis. This block, in the fingerprinting testbed, runs a software-defined GNSS receiver, gnss-sdr [26], processing signal samples from each one of the files, generating three main results:

- Observable RINEX files
- Prompt correlator IQ values in binary file.
- Tracking raw information for the calibration signal.

The gnss-sdr was developed by the Centre Tecnològic de Telecomunicacions de Catalunya (CTTC) in Castelldefels, Barcelona, Spain. The used version was the version 0.0.18.git-next-6b9ad0332.

Further details related to the calibration signal can be found in Section 3.5.1.

Here, the work of the metrics collector block is more challenging, as the pseudoranges will be available every second, and the output of the prompt correlator will be available every 4 ms for E1. Moreover, for E5a, a sample will be available every 20 ms. Hence, it is necessary to decimate values between samples and provide one output every second, aligned with the slowest data source, in this case, the pseudoranges.

It is actually possible to increase the frequency of the pseudorange outputs, but it is impractical to modify the integration periods of the correlators. The time alignment of this block is carried out in the “Metric collector” block, which provides outputs aligned with the slowest component, the pseudoranges. Time shift and alignment are also required in this block, as the $\bar{BI}$ metrics are computed once a pseudorange arc is long enough to provide proper reliable $\bar{BI}$ metric estimates. Therefore, that has to be accounted for when aligning metrics in the output of the PVT engine block, introducing a global delay in the output for real-time applications.

It is to be noted that while this block in the testbed is operating in an offline mode, in order to enhance the testbed to allow near-real-time operations, the following changes and updates are needed:

- Configure/modify gnss-sdr to obtain the samples directly from a network socket (the implementation in the testbed is implemented reading files). The datastreams shall be forked among the raw signal processing block and the PVT engine block.
- Configure/modify gnss-sdr to provide outputs to the “IQ plane analysis”, likely via network sockets, allowing the implementation of the internal blocks of the system to communicate among each other using a normal Ethernet network (this also facilitates the distribution of the system blocks into different computing nodes).
- Implement a function inside the “$\bar{BI}$ estimates” to estimate the wind-up effect (as currently the windup effect is not simulated in the signals generated in the laboratory to simplify the implementation).

**Figure 6:**
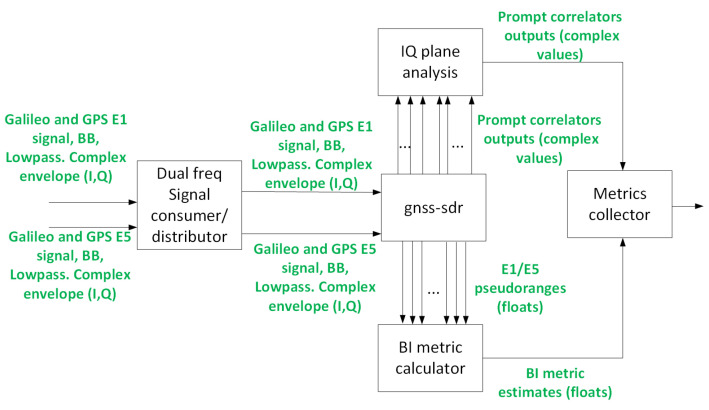
PVT engine block.

**Figure 7:**
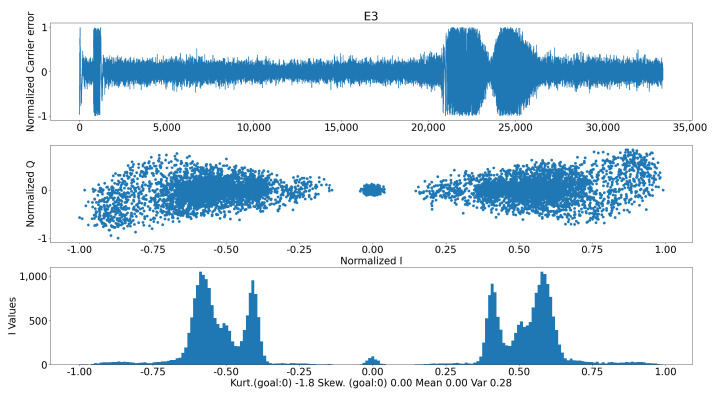
IQ plane with spoofing. These results are in line with those shown in [7].

Gnss-sdr was not modified for the testbed, it was merely integrated. The PVT engine can be seen in Figure 6. As mentioned in Section 2.2.1, in the presence of a synchronous (even an SCER one) attack, we will find some dispersion in the in-phase axis that can indicate the presence of an attack. Such a situation, derived from the datasets generated in the DLR laboratory (see Section 3.2), can be seen in Figure 7, where a spoofer is present for Galileo satellite E03. Four Gaussian PDFs can be clearly identified in the lower pane of the figure, indicating that two sources with Galileo-E03-like waveforms are encoding symbols into them. Of course, one should take care of the fact that multipath may also cause similar patterns; hence, the combination of several types of metrics and the usage of ML techniques for the detection are recommended.

**Figure 8:**
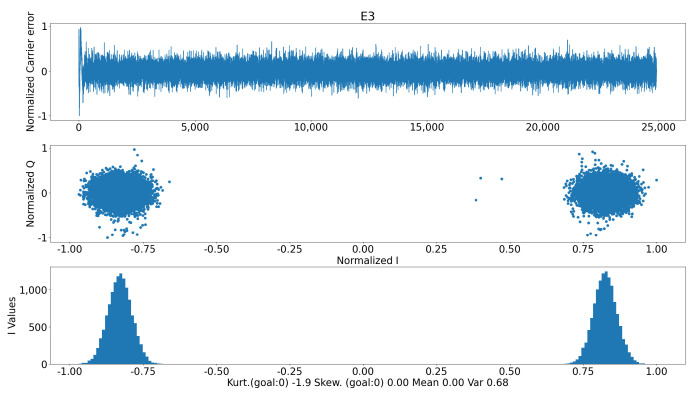
No spoofing IQ plane.

In Figure 8, there is a similar situation where no spoofing signal (while tracking the signal) is observed. This is an important point to carefully account for in order to reduce the amount of false positives: the model shall only be exposed to samples of the IQ plane if the signal is being properly tracked and the carrier error is low. This will imply the usage of splines to interpolate future values should the carrier error be too high or repeat old values or not output data for that particular satellite into the ML block (Section 3.6).

This block is also in charge of computing the $\bar{BI}$ metric defined in Section 2.1.1.

#### 3.5.1. Calibration of the Receiver Delay for $\bar{BI}$ Metric Computation

It is mandatory, in order to remove the effects of the electronics receiver (i.e., the differential delay between the two channels (E1 and E5) caused by the receiver used), to include a GNSS-like signal in the input of the receiver electronics. This was performed by removing the satellite E30 from the simulated Galileo constellation and including a Galileo-like waveform, both in E1 and E5 for satellite E30, with no navigation message.

This last point, the lack of a navigation message, is a very relevant point, as this means the GNSS receiver (in this case, the gnss-sdr) will not be able to use this satellite for PVT as it will not be able to align the estimated pseudoranges with the others, as there will not be Galileo time information included in the navigation message.

In order to overcome this problem, the E30 estimated phase difference and code difference between both channels are computed in a different manner for the calibration signal.

It is to be noted here that with the calibration signals, we are not operating with pseudoranges as such, because this type of observable is estimated after decoding the Galileo system time (GST) of the time of transmission, and, in our case, we do not have a valid navigation message.

The goal of the calibration signal is to estimate the delay, both for code and phase, between both channels. Then, the following operation is required to perform the computation:

- For phase: access the E30 tracking block PLL (part of the tracking block of gnss-sdr in Figure 6) and extract the new carrier phase estimated for both channels (E1 and E5).
- For code: access the tacking stage DLL (part of the tracking block of gnss-sdr in Figure 6); then, extract the new PRN delay estimates for both channels (E1 and E5).

Such values can be extracted easily from the gnss-sdr due to the binary-files-dumping capability that the SW has (supporting both Octave format and a binary ad hoc format). Such ad hoc binary format provides the information in C-format, as per Table 2.

Other debugging information is also available in these files.

The calibration (coming from the calibration signal of E30) shall be applied to each one of the RINEX phase and geometry-free estimates (for the other satellites) individually.

Access to the sample counters and alignment to the timestamp associated with the beginning of the tracking are needed per channel and satellite, for proper time alignment at this stage. In our case, a priori knowledge of the timestamps at the beginning of each signal record was available. For a real-time scenario, the computation shall be carried out on the fly and, of course, a common oscillator shall be fed to both front-ends.

Once both values are available, derived from the calibration signals injected at the receiver input, the delay between each channel caused by the receiver can be computed both for code and phase.

### 3.6. Machine Learning Block

This block shall, first of all, do the final time alignment between the outputs of the two raw signal processing blocks and the PVT engine block, ensuring that all the metrics are properly time-aligned and sampled at the lowest frequency (i.e., 1 Hz, due to the pseudoranges availability).

Then, all the metrics are provided to previously trained machine learning algorithms, which, in turn, predict the presence of Spoofers in the input signals. In order to train such models, data from a simulator (as we carried out in Section 3) can be used, providing the models with a set of meaningful features with and without spoofers and jammers. In real operational deployments, data from real attacks can be used to move from an initial status, with models trained solely with simulated data, to models trained with a combination of simulated and real attacks.

For the operational version of the system tested in the testbed, careful coding and signaling management is required to ensure that the outputs of the different blocks are treated properly. Taking into account that the spoofing attacks may elapse for several minutes in order to ensure deviating the victim to a point of interest, ensuring a subsecond accuracy when aligning the real-time metrics may not be mandatory. Moreover, the values of the metrics could be managed in a way that provides a level of confidence with respect to the presence of the spoofer. Indeed, decision-tree-based ML algorithms are a natural way of providing such a framework. This is because the decision-tree-based ML algorithms have a natural hierarchical structure and work with conditional probability. Each path from the starting root to a leaf node is a specific sequence of decisions based on feature obtained values. This path can be seen as a conditional probability sequence where each decision point narrows down the spoofer-presence likelihood by considering additional evidence (i.e., more metrics with values that point to the presence of the spoofer).

This block should load a pre-trained ML model. Such a model shall be the one that provides the best accuracy, with a particular hyper-parameter configuration. Specifically, this applies to the models analyzed in this paper:

- Decision Trees: minimum number of samples required to split into an internal node and the maximum depth of the tree.
- Random Forest: minimum number of samples required to split into an internal node and the maximum depth of the tree.
- Ada Boost: Number of estimators.
- Convolutional Neural Networks: Activation function and solver.

### 3.7. Testing Dataset Description

The testing dataset was generated using the advanced equipment from DLR described in Section 3.2 (MASTER). This allows for ensuring a maximum similarity to a real scenario with real spoofers.

The goal of the scenarios was to simulate a set of conditions with and without spoofers and jammers with and without atmospheric conditions impacting the measurements. All the scenarios were simulating a victim antenna on 2022-07-28T12:00 (UTC), in longitude = 11.27776 deg and latitude = 48.08479 deg.

For that purpose, the following scenarios were generated:

- Atmospheric effects ON (troposphere and ionosphere).
- Atmospheric effects OFF (troposphere and ionosphere).

For both options, a dynamic attack was simulated. It consisted of a first attack of RFI, followed by a spoofing signal that started by increasing its power and, finally, overpowering the real Galileo satellite signal.

In order to properly train the ML models, the same scenarios were generated with and without spoofers and jammers.

The used sampling frequency was 20 MHz for both E1 and E5, the digitization was performed using 14-bits for I and another 14-bits for Q. GPS and Galileo were simulated, although the work focused mainly in Galileo. It should be noted that no multipath was considered in our tests.

Both the spoofed and genuine signals (non-spoofed ones) were generated by the Spirent GSS9000 units of the MASTER facility; all Galileo and GPS satellites were spoofed in the spoofed datasets. Both authentic signals and spoofed ones were present when the spoofing was active. The datasets were divided between spoofing active (spoofing ON) and spoofing not active (spoofing OFF), then fed into the proposed solution (that included the supervised machine learning algorithms). When the spoofing was active, the simulation was the one with a static-victim scenario. The first 30 s of the simulations included only authentic signals (these 30 s were also fed as spoofing OFF scenarios to the machine learning algorithms). After 30 s, the spoofing signals appear and start to rise in power. At 90 s of simulation time, the spoofing signals reach and overpower by 3 dBs the authentic signals. At that point the power rise stops, and the position produced by the spoofing signals starts to move away from the authentic one, first very slow but after 10 s, with a speed of 20 m/s.

The dataset included samples with atmospheric effects, both ON and OFF, to enhance the generalization of the machine learning (ML) model. This approach accounts for cases where atmospheric effects over Germany were present and those where these were nonexistent. When atmospheric effects were enabled, the following configuration was applied to the Spirent-generated signals: the tropospheric delay model used was the STANAG model, based on the NATO Standard Agreement STANAG 4294 [27]. The surface refractivity index was set to 324.8, ensuring accurate modeling of atmospheric refraction effects. The STANAG model shares similarities with the IS-GPS-200 specification [28].

For ionospheric delay, the Klobuchar model was employed to simulate differential delays. This included the Alpha and Beta parameters provided for both RF signals. The parameter values [29] ranged from 4.656612×10−9 to −5.96046×10−8 seconds/semicircle for Alpha, and from 79,872 to −393,216 s for Beta. The relevant constellation-dependent data for navigation messages were computed.

The satellite hardware imperfections were modeled to be based on real navigation message values for the real constellation, while the spoofed ones had ideal values (i.e., BGD of 0 ns). This imposes clear differences in values when it comes to the simulated scenarios. This will be further evaluated in future work, particularly for dual-frequency attacks, as outlined in Section 2.1.1.

These datasets, generated by the MASTER facility, were used for the testing and training, divided as described in Section 3.7.1.

#### 3.7.1. Cross-Validation Results

In a similar manner to the work developed in [3] for the CMCU [30], K-folds were used, with K = 5. From the overall amount of data samples, 30% of them were used for validation. The other 70% were used to train the models, using the K-folds technique, dividing the dataset into five groups (K = 5). The choice of using 5-fold cross-validation (i.e., K = 5) is based on findings from prior researchers, notably the study by Kohavi [31]. In this work, the authors provide a detailed evaluation of fold numbers in K-fold cross-validation, examining their impact on accuracy estimation bias. The study indicates that the performance estimates become more stable starting at K = 5. Therefore, to balance computational efficiency with reliable results, we selected K = 5 as the optimal value for this application.

The complete dataset, prior to the splitting, consisted of 765,664 points (including all cases).

### 3.8. Results

Table 3, Table 4, Table 5 and Table 6 show the performance classifications for different machine learning classification algorithms, resulting from the MASTER’s generated datasets. As implied by Table 4, the Random Forest (minimum sample of 2, maximum depth of 66 layers, 10 trees) outperforms the other ML methods for the presented method, with an obtained probability of missed detection (PMD) of $PMD<7.743\times 10^{-6}$ and a probability of false alarm (PFA) of $PFA<9.62\times 10^{-6}$. As for the decision trees (Table 3) with a minimum number of samples for splitting an internal node of 2 samples and maximum depth of 263 layers, the obtained PMD and PFA are: $PMD=2.32\times 10^{-4}$ and $PFA=2.60\times 10^{-4}$. In the case of the Ada Boost (Table 5), with a maximum number of estimator of 1900, we obtained $PMD=6.12\times 10^{-4}$ and the $PFA=6.54\times 10^{-4}$. As for the convolutional neural networks (CNN), with 459 layers, using as activation function the arc-tangent function, and the adaptive moment estimation (Adam) as the solver, the results were very poor, providing a PFA of 36.53% and a PMD of 25%; hence, its usage for the next steps in the implementation of a real-time system using these metrics and ML will be disregarded. With regards to the accuracy of the CNNs, this is in line with other authors, as will later be detailed in Section 3.9. This result should not be surprising as it is known that neural networks do not usually perform well for datasets that are small (being outperformed in those cases by models that have a smaller tendency toward over-fitting). As pointed out in [32], there should be a trade-off between the fitting capacity of the model to be used and the complexity of the presented problem and dataset. As the applied K-folds technique in the present work implies the model will face data that were never shown to it during the training phase, models with known tendency to over-fitting lead to poor results with small subsets. This does not mean NN could not potentially be used for this purpose, but the datasets for training will need to be considerable bigger, helping NN not to fit to irrelevant features or noise. On top of this, it is possible that as the data fed into the ML algorithms are generated at several stages of the receiver, the metrics are highly correlated, which may require further normalization [32] to use NNs to their maximum capacity, as CNNs struggle with certain data structures.

The four supervised methods were selected based on the dataset’s dimensionality, the nature of the problem, and a primary goal of minimizing computational demands. Additionally, these classical methods allow for extensive comparison with the existing literature, providing a solid foundation for evaluating results. Other algorithms, for instance, deep learning approaches, while powerful, were not pursued due to their computational complexity and the moderate size of the dataset, which is better suited for non-neural network methods, as the tests with CNN results further suggest.

Table 7 summarizes the found results. As can be seen, the best results are obtained with the random forest algorithm.

### 3.9. Comparison with Other State-of-the-Art Solutions

The reported error rate is calculated as 100%− *accuracy*, where accuracy represents the percentage of correct classifications by the algorithm (i.e., the ratio of correct classifications to the total number of samples, expressed as a percentage).

As can be seen in Table 8, the provided results are remarkable for the solution with random forest. As for the CNN results, these are in-line with the findings of other researchers, applying other metrics to CNN and even deep learning, as outlined in [9]. When comparing the state-of-the-art results, it should be noted that our results do not consider a mobile victim receiver. Further work will be developed in the future in the mobile environment context.

### 3.10. Considerations Towards Mobile Receivers

Some theoretical considerations about the extension of this proposed methodology towards mobile receivers are discussed in this section.

The context of a mobile victim implies different challenges for the attack and for the protection of the victim.

Both skew and kurtosis metrics will not be severely impacted by the mobile nature of the victim and/or the attacker. This is because the goal of both metrics is to detect the jamming signals, typically prior to the start of a spoofing attack.

With regards to the TKEO metric, should the victim and spoofer keep a continuous distance, or in case the spoofer is always able to keep the same channel characteristics between the victim and the spoofing transmitting antenna, then no major effect should be expected. On the other hand, in case the spoofer and the victim are not keeping a continuous distance or in case the channel between both is not behaving in a controlled manner (i.e., spoofing signal complete or partial blockage), the TKEO metric will show patterns that may not match the non-spoofed scenarios. This implies that even in the worst case scenario, the impact is not negative for the detection, quite the opposite. Moreover, new metrics for ML could be derived from this effect. For this, the ML models need to be exposed to the time evolution of the TKEO metric, so some upgrade may be required to the proposed technique.

The symbol dispersion impact will depend greatly on the control with regards to the estimation of the victim movement that the spoofer will have. Should the estimate not be good enough, then the control of the synchronization of the attack (i.e., the capability the spoofer may have to align the fake signals with the real ones in the antenna phase center of the victim’s antenna) will not be possible. The effect in the in-phase symbol dispersion depends a lot on this overlapping, so only in case the attacks successfully estimate the distance between the attacker’s transmitting antenna and the victim’s receiver antenna will this be possible. It is understood that the spoofer will try to start the attack in a synchronous manner in order to reduce the effect in the victim’s tracking loops. Hence, the mobile environment provides the attacker with a clear challenge. In case the attacker opts for jamming the victim’s receiver prior to the start of the attack (so the tracking loop effects will not be so evident) the analysis on the skew and kurtosis will detect the jamming signals, making the victims aware of the possible incoming attack (and, indeed, the decision trees are very good to model the increase of attack probability, due to its nature).

Finally, it is the metric based on the satellite instrumental delay that may experience more challenges in the mobile context. The main reason is that in order to compute the undifferentiated carrier-phase ambiguity-free combination (BI) as the computation of the averaged difference value of the code and phase geometry-free for an entire tracked satellite arc, the more continuous the arc is, the more accurate the estimate will be. A mobile environment channel is expected to provide many blockages, which will imply starting a new tracking process, making such arcs not as stable as a fixed victim receiver case. This limitation is currently being analyzed in order to overcome it in future versions of the solution.

## 4. Conclusions

Spoofing and jamming attacks represent a very serious threat for systems relying on GNSS (e.g., maritime and aeronautical systems). To fight against that risk, some authentication techniques, like NMA in Galileo, are available. However, this may not be enough to counteract the spoofing attacks based on SCER. For this particular case, a complementary solution based on the application of machine learning methods to the receiver search space has been introduced in this paper. This type of solution could also be interesting not only for protecting against SCER attacks but also for systems protecting critical infrastructure (CI) or as a tool for spectrum regulatory authorities to ensure that no illegal activities are being performed across wide geographical areas.

Some fingerprinting metrics were analyzed, and one new metric, based on satellite group and phase delay, was proposed for its usage in spoofing detection. A system demonstrator was implemented and tested. The tests were performed using the DLR GNSS signals advanced laboratory (MASTER).

The obtained results show promising results using ML, for random forest, with a $PMD<7.743\times 10^{-6}$ and a $PFA<9.62\times 10^{-6}$. These results are similar to those of the central machine learning computation unit (CMCU) [30] and provide a very competitive solution for protecting CIs and monitoring wide areas against illegal GNSS transmissions (i.e., jamming and spoofing).

The CNN provided poor results with our proposed metrics. Nonetheless, these results are in line with the accuracies obtained by [9], with CNNs and with deep learning. Nonetheless, future work will be applied to enlarge the datasets and use further normalization techniques [32] to improve the obtained performance.

The work will now continue to implement a real-time system based on the current workbench. This will include the implementation of the computation engine for some of the effects estimations that are assumed to be computed in Section 2.1.1 and were made zero in the simulations (e.g., wind-up effect). Several methods, for instance, the one proposed in this paper and the CMCU [3,30], could be further combined by using an upper layer machine learning. Moreover, other protection systems at other stages of the GNSS receiver could be further combined, like the GALANT antenna array [4]. These combinations of detection systems make the quick detection of jamming and spoofing attacks quicker and more accurate. On top of that, other metrics, related to the channel estimation, will be included in the fingerprinting features that are fed into the ML algorithms, such as the line-of-sight-factor (LOSF) [33].

Further work will include deeper testing of the solution, as no case of single-frequency E1 spoofing was performed, and only dual-frequency spoofing was always used. As discussed in Section 2.1.1 this actually makes the detection harder for the proposed system, so it is expected that single-frequency attacks will further improve the results. This will also imply further evaluation of the imperfection modeling to further test the protection that the solution can offer for dual-frequency SCER attacks, although, as outlined in Section 2.1.1, the main goal of the BGD metric will lay on the detection of single-frequency attacks or attacks where the spoofer fails to control channel coherency, providing very different values from those of the real satellites.

**Figure 9:**
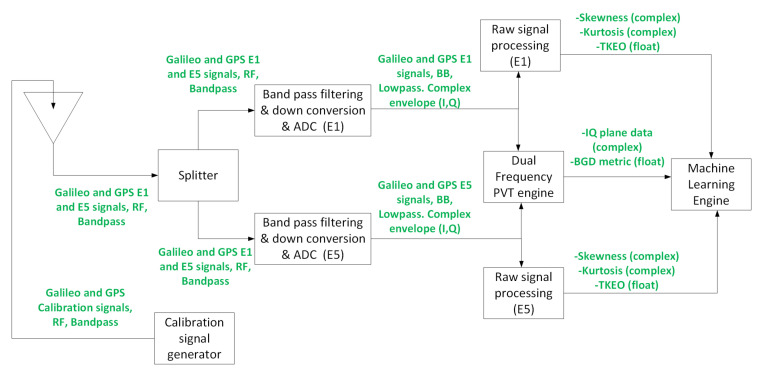
Initial design of the first prototype of the detection system, the development, based on the results of the testbed presented here will be part of the future work.

Of course there will be some differences between the existing testbed and the first prototype of the system. The overall architecture of the testbed was explained in Section 3, and the initial design of the first prototype of the final system can be found in Figure 9.

The main differences between the architecture in Section 3 and the one in Figure 9 is that the MASTER facility is removed and replaced by a GNSS antenna, and a separate calibration signal generator is included. Changes in the GNSS receiver inside of the dual frequency PVT engine will also be required to operate in real time and compute the required metrics in a continuous manner.

The analysis on how to overcome the BI estimate in real-time will continue, along with the adaptation of the metric for satellite instrumental delay adaptation to mobile environments.

Another natural continuation of the work developed in this system is its application to detect known GNSS ground or space segment anomalies. Furthermore, using non-supervised models makes the detection of outliers (which may be related to system anomalies) easier. Note that in the present work, we did not identify each GNSS satellite in the fingerprinting but just flag the presence of jamming and spoofing signals.

Another possible continuation will be including the analysis of skew and kurtosis of the prompt correlator output instead of using large amount of normalized IQ plane time series.

The pseudoranges analysis, detecting outliers, and the comparison of the decoded navigation messages with an external reference (e.g., Internet sources) or the timing error between the computed PVT and a trusted time reference (e.g., atomic clocks) could provide enhanced security. If the receiver is a fixed one, then monitoring the PVT error is also recommended. All of these are straightforward to implement.

## Figures and Tables

**Table 1:** State of the art techniques comparison.

Reference	Used Features	Used ML Algorithm	Reported Accuracy
W. Wang et al. [5]	IQ imbalances, ADC non-linearities, phase noise, P.A. non-linearities	SVM	0.99
M. Foruhandeh et al. [7]	IQ samples	MVN	>0.99997
A. Iqbal et al. [8]	SQM	TS-ANN	0.993
A. Iqbal et al. [8]	SQM	K-NN	0.995
C. Guo et al. [9]	STFT	RESNET-50 (Deep Learning)	0.539
C. Guo et al. [9]	IQ samples	CNN	0.650
Q. Jiang et al. [10]	GNSS Search space	VAE	0.957
X. Zhang et al. [11]	IQ samples	CAE and noise-like generator (NACA)	0.997

**Table 2:** Ad hoc binary format provides the following information in C-formatted data.

Value	C Data Type
ABS VE correlator	Float
ABS Early correlator	Float
ABS Prompt correlator	Float
ABS Late correlator	Float
ABS VL correlator	Float
Prompt correlator in-phase	Float
Prompt correlator quadrature	Float
Start sample of the tracking for PRN	Uint64
Accumulated carrier phase [rad]	Float
Carrier Doppler [Hz]	Float
Carrier phase rate [Hz/S]	Float
Code frequency [Hz/S]	Float
Code frequency rate [Hz/$S^{2}$]	Float
Carrier error [Hz], output PLL discr.	Float
Carrier error [Hz], output PLL filter	Float
Error code [# of chips]	Float
Filtered error code [# chips]	Float
CN0 estimates [dB/Hz]	Float
Carrier lock test	Float
Remanent code phase [Samples]	Float
PRN	Uint32

**Table 3:** K-folds results.

Decision Tree. minsamp = 2, maxdepth = 263		
	**True Class: Spoofer Present**	**True Class: Spoofer Not Present**
Classified as Spoofer Present	129,125	27
Classified as Spoofer Not Present	30	103,900

**Table 4:** Random Forest results.

Random Forest. minsamp = 2, maxdepth = 66		
	**True Class: Spoofer Present**	**True Class: Spoofer Not Present**
Classified as Spoofer Present	129,152	0
Classified as Spoofer Not Present	0	103,930

**Table 5:** Ada Boost results.

Ada Boost. Estimators: 1900		
	**True Class: Spoofer Present**	**True Class: Spoofer Not Present**
Classified as Spoofer Present	129,084	68
Classified as Spoofer Not Present	79	103,851

**Table 6:** Convolutional Neural Networks results.

CNN. 459 layers, Activation function arctan, Sol.Adam		
	**True Class: Spoofer Present**	**True Class: Spoofer Not Present**
Classified as Spoofer Present	85,780	43,372
Classified as Spoofer Not Present	28,587	75,343

**Table 7:** Overall results for the different AI algorithms with the proposed methodology.

Overall Results		
**ML Model**	**PFA**	**PMD**
Random Forest	<9.62 $\times 10^{-6}$	<7.743 $\times 10^{-6}$
Dec. Tree	2.60$\times 10^{-4}$	2.32$\times 10^{-4}$
ADA Boost	6.54$\times 10^{-4}$	6.12$\times 10^{-4}$
CNN	0.37	0.25

**Table 8:** Comparison of the accuracy obtained with the proposed solution (with random forest) and other state-of-the-art research.

Comparison with State of the Art	
**Methodology**	**Error Rate** (100%−accuracy)
Deep Learning (RESNET50), STFT [9]	46.1%
K-NN, SQM [8]	35%
CNN, direct IQ [9]	35%
VAE, Search space [10]	4.32%
CAE, Noise-like reconstruction [11]	0.3%
SVM, several RF features [5]	0.01%
MVN, direct IQ [7]	<$2.9\times 10^{-5}$%
The methodology proposed in this paper	<$4\times 10^{-6}$%

## Data Availability

Data are contained within the article.

## References

[1] Humphreys T. (2013). Detection Strategy for Cryptographic GNSS Anti-Spoofing. IEEE Trans. Aerosp. Electron. Syst..

[2] Radoš K., Brkić M., Begušić D. (2024). Recent Advances on Jamming and Spoofing Detection in GNSS. Sensors.

[3] Gallardo F., Yuste A.P. (2020). SCER Spoofing Attacks on the Galileo Open Service and Machine Learning Techniques for End-User Protection. IEEE Access.

[4] Konovaltsev A., Marcos E., Cuntz M., Meurer M., Buesnel G., Lange W. Development of Array Receivers with Anti-Jamming and Anti-Spoofing Capabilities with Help of Multi-Antenna GNSS Signal Simulators. Proceedings of the 32nd International Technical Meeting of the Satellite Division of The Institute of Navigation (ION GNSS+ 2019).

[5] Wang W., Lohan E.S., Sanchez I.A., Caparra G. Pre-correlation and post-correlation RF fingerprinting methods for GNSS spoofer identification with real-field measurement data. Proceedings of the 2022 10th Workshop on Satellite Navigation Technology (NAVITEC).

[6] Wang W., Aguilar Sanchez I., Caparra G., McKeown A., Whitworth T., Lohan E.S. (2021). A Survey of Spoofer Detection Techniques via Radio Frequency Fingerprinting with Focus on the GNSS Pre-Correlation Sampled Data. Sensors.

[7] Foruhandeh M., Mohammed A., Kildow G., Berges P., Gerdes R. Spotr: GPS Spoofing Detection via Device Fingerprinting. Proceedings of the 13th ACM Conference on Security and Privacy in Wireless and Mobile Networks.

[8] Iqbal A., Aman M., Sikdar B. (2023). Machine and Representation Learning Based GNSS Spoofing Detectors Utilizing Feature Set From Generic GNSS Receivers. IEEE Trans. Consum. Electron..

[9] Guo C., Yang Z. A Robust RF Fingerprint Extraction Scheme for GNSS Spoofing Detection. Proceedings of the 36th International Technical Meeting of the Satellite Division of The Institute of Navigation (ION GNSS+ 2023).

[10] Jiang Q., Sha J. (2024). Radio Frequency Fingerprint Identification Based on Variational Autoencoder for GNSS. IEEE Geosci. Remote Sens. Lett..

[11] Zhang X., Huang Y., Tian Y., Lin M., An J. (2023). Noise-Like Features-Assisted GNSS Spoofing Detection Based on Convolutional Autoencoder. IEEE Sens. J..

[12] European Commission (2016). European GNSS (Galileo) Open Service Signal-in-Space Interface Control Document.

[13] Wu W., Guo F., Zheng J. (2020). Analysis of Galileo signal-in-space range error and positioning performance during 2015–2018. Satell. Navig..

[14] EUSPA Galileo Satellites Metadata. https://www.gsc-europa.eu/support-to-developers/galileo-satellite-metadata.

[15] Rovira Garcia A., Juan J., Sanz J., Gonzalez-Casado G., Ibáñez D. (2015). Accuracy of ionospheric models used in GNSS and SBAS: Methodology and analysis. J. Geod..

[16] Li Z., Yuan Y., Li H., Ou J., Huo X. (2012). Two-Step Method for the Determination of the Differential Code Biases of COMPASS Satellites. J. Geod..

[17] Nacer Naciri S.B. (2020). Multi-GNSS Ambiguity Resolution For Signal Obstruction in PPP. Inside GNSS. https://insidegnss.com/multi-gnss-ambiguity-resolution-for-signal-obstruction-in-ppp/.

[18] Seco-Granados G., Gómez-Casco D., López-Salcedo J.A., Fernandez-Hernandez I. (2021). Detection of Replay Attacks to GNSS based on Partial Correlations and Authentication Data Unpredictability. GPS Solut..

[19] Psiaki M.L., Humphreys T.E. (2016). GNSS Spoofing and Detection. Proc. IEEE.

[20] (2022). GNSS Software Receivers.

[21] Blum R., Dütsch N., Dampf J., Pany T. Time Synchronized Signal Generator GNSS Spoofing Attacks against COTS Receivers in over the Air Tests. Proceedings of the 2021 International Technical Meeting of The Institute of Navigation.

[22] Huang L., Lu Z., Ren C., Liu Z., Xiao Z., Song J., Li B. (2022). Research on detection technology of spoofing under the mixed narrowband and spoofing interference. Remote Sens..

[23] Zwillinger D., Kokoska S. (2000). CRC Standard Probability and Statistics Tables and Formulae.

[24] Navidi W. (2011). Statistics for Engineers and Scientists.

[25] Solnik S., Rider P., Steinweg K., DeVita P., Hortobagyi T. (2010). Teager–Kaiser energy operator signal conditioning improves EMG onset detection. Eur. J. Appl. Physiol..

[26] Fernández–Prades C., Arribas J., Closas P., Avilés C., Esteve L. GNSS-SDR: An Open Source Tool For Researchers and Developers. Proceedings of the 24th International Technical Meeting of the Satellite Division of the Institute of Navigation.

[27] Office N.S. (1991). NATO Standard Agreement STANAG 4294.

[28] Office N.G.J.P. (2021). IS-GPS-200: Navstar GPS Space Segment/Navigation User Interfaces.

[29] Klobuchar J.A. (1987). Ionospheric time-delay algorithm for single-frequency GPS users. IEEE Trans. Aerosp. Electron. Syst..

[30] López F.G., Yuste A.P. (2021). Operational Deployment of GNSS Anti-spoofing System for Road Vehicles. Proceedings of the Communication Technologies for Vehicles: 16th International Workshop, Nets4Cars/Nets4Trains/Nets4Aircraft 2021, Madrid, Spain, 16–17
November 2021.

[31] Kohavi R. (1995). A Study of Cross-Validation and Bootstrap for Accuracy Estimation and Model Selection. Proceedings of the 14th International Joint Conference on Artificial Intelligence.

[32] Goodfellow I., Bengio Y., Courville A. (2016). Deep Learning.

[33] Yuste A.P., Llumiquinga Pachacama J., García J.S. Characterization of the 2.4 GHz-band using a semiempirical model and a ray tracing model. Proceedings of the 2023 IEEE Conference on Antenna Measurements and Applications (CAMA).

